# Digital breast tomosynthesis in breast cancer screening: an ethical perspective

**DOI:** 10.1186/s13244-024-01790-w

**Published:** 2024-08-26

**Authors:** Simon Rosenqvist, Johan Brännmark, Magnus Dustler

**Affiliations:** 1 Department of Philosophy, Linguistics and Theory of Science, University of Gothenburg, Göteborg, Sweden; 2 Department of Philosophy, Stockholm University, Stockholm, Sweden; 3 Diagnostic Radiology, Department of Translational Medicine, Faculty of Medicine, Lund University, Lund, Sweden; 4 Medical Radiation Physics, Department of Translational Medicine, Faculty of Medicine, Lund University, Lund, Sweden

**Keywords:** Breast, Digital mammography, Digital breast tomosynthesis, Ethics, Health policy

## Abstract

**Abstract:**

Although digital breast tomosynthesis has higher sensitivity than digital mammography and at least as high specificity, digital mammography remains the most common method for conducting mammographic screening. At the same time, mammography systems are now delivered “DBT-ready” and can be used for either digital mammography or digital breast tomosynthesis. In this paper, we ask whether it is ethically permissible to use such equipment for digital mammography, given its lower sensitivity. We argue it is not, and that clinics are ethically required to use their DBT-ready equipment to screen with digital breast tomosynthesis whenever this is practically possible. Our argument relies on a comparison between digital breast tomosynthesis and a hypothesized improvement in the image quality of digital mammography.

**Critical relevance statement:**

Women may lose out on the benefits of screening with digital breast tomosynthesis when DBT-ready equipment is used to screen with digital mammography; we argue that this practice is ethically problematic.

**Key Points:**

- Digital breast tomosynthesis finds more cases of breast cancer than digital mammography.
- Mammography equipment can often be used to screen with both digital breast tomosynthesis and digital mammography.
- When they can, clinics are ethically required to use existing equipment to screen with digital breast tomosynthesis instead of digital mammography.

## Introduction

Digital mammography (DM) is the most common method for conducting mammographic screening. However, an alternative now exists in the form of digital breast tomosynthesis (DBT), which has higher sensitivity and at least as high specificity as DM [1]. The continued use of DM in screening has led to an ethically complicated situation, as modern mammography equipment is delivered “DBT-ready” and can be used for both DM and DBT. When clinics use such equipment to screen with DM instead of DBT, they, in effect, choose a less sensitive method of examination. For this approach to be ethically acceptable, there must exist a clear and scientifically supported advantage to the use of DM in these situations, which does not generally seem to be the case.

In this paper, we argue that when practically possible, clinics are ethically required to use DBT-ready equipment to screen with DBT instead of DM. Our argument relies on a comparison between DBT and a hypothesized improvement in the sensitivity of DM (“DM+”). The thought experiment is designed to raise structurally similar issues as DBT, but with less potential for status quo bias to affect our judgment. Importantly, the paper does not attempt to address lingering scientific questions about whether and how DBT contributes to overdiagnosis (detecting tumors that would not otherwise have caused symptoms) or reduces mortality. Instead, it is argued that due to the higher sensitivity of DBT, we are ethically required to use it with DBT-ready equipment until these scientific questions have been more fully addressed.

## A thought experiment

Imagine that a new type of digital detector has substantially improved the image quality of DM, increasing its sensitivity without affecting its specificity. The technology becomes known as DM+ and manufacturers begin to use it exclusively in their product lines. These new DM+ systems are now replacing worn-out equipment at a major hospital. In charge of the hospital’s mammography unit is a senior physician who has closely followed the development of DM+. He decides that when installing the new systems, a software filter should be activated that artificially reduces the image quality to that of ordinary DM images. The software filter was originally included for research and testing purposes and is considered highly reliable. Due to the use of this filter, the mammography unit goes on to detect cancers at roughly the same rate as before.

At this point, a junior radiologist takes the stage. She says it was ethically wrong to implement the filter because the lower sensitivity might result in unnecessary deaths. After learning of the situation, the hospital director summons the senior physician. “Why would you restrict the image quality of the new systems?” the director asks. But the senior physician is unfazed by the attention and explains his reasoning as follows:

I agree that DM+ imagery improves sensitivity at no loss of specificity compared to DM—there is broad scientific consensus on this point. However, we do not know for certain that detecting these additional cancers will benefit patients. For example, we lack data on how DM+ images could contribute to overdiagnosis, and there are presently no studies investigating the effects on mortality. I admit we have not found much of a difference in the characteristics of cancers discovered with DM+ and DM, and one study even suggests that DM+ detects more relevant and dangerous tumors than DM. However, it remains true that DM+ might not improve survival and might increase the rate of overdiagnosis compared to DM. Before we use the new higher-quality images, we should wait for more conclusive data on interval cancer, preferably from sufficiently powered randomized studies. When better data is available in 5- or 10-years’ time, we can reconsider whether to turn off the software filter.

The senior physician and the junior radiologist share their estimates of the sensitivity and specificity of DM and DM+. They are equally cognizant of the risks and negative consequences posed by overdiagnosis. What they disagree about is not the science but how to act in light of it. The senior physician believes that greater certainty is needed, because in medicine, “good” or “conclusive” evidence for patient benefits is required. He therefore believes we should wait for further studies. The junior radiologist agrees these studies should be conducted but believes the new images should be used while we wait for the results. She believes that what matters is not conclusive evidence for patient benefits but expected or probability-adjusted value for patients. As she sees it, although there is a risk that DM+ is inferior to DM, it is outweighed by the chance that DM+ is superior to DM.

We believe most of us will side with the junior radiologist. The senior physician is right to take questions of overdiagnosis and mortality seriously, but such concerns do not justify reducing the sensitivity of DM+ equipment.

If it is wrong to restrict the image quality of DM+, why is that so? Likely because we assume that, everything else being equal, increasing the sensitivity of ordinary digital mammography will benefit patients. This explains why modifications to DM that merely increase sensitivity, such as improving tissue contrast or image resolution, will be promptly implemented.

Since we assume that, everything else being equal, increasing the sensitivity of DM will benefit patients, the senior physician must demonstrate that everything else is not equal. We therefore require evidence that, for example, DM+ detects less dangerous tumors or has lower specificity than DM. But as the case is constructed, no such evidence is available, which is why the senior physician’s decision appears unreasonable.

Finally, note that appealing to inequality is unlikely to help the senior physician’s position. While the fictional DM+ equipment is not yet owned by all hospitals, it will be universally available in the future, since all new mammography systems are manufactured with the novel detectors. Such temporary inequality is less problematic than a permanent one.

## DBT in screening

Although the case of DM+ is fictional, it shares important features with digital breast tomosynthesis (DBT), which is a form of digital mammography that started development in the 1990s and has been commercially available since the early 2010s [2]. Whereas DM has the x-ray system capture two-dimensional images from a stationary position, DBT instead captures a series of images while moving the tube on a circular arc. These images are then combined algorithmically into a pseudo-3D image, which enables readers to see past obscuring tissue.

Since DBT can discover cancers that are hidden from DM, it has been seen as a promising way to improve the sensitivity and specificity of traditional mammography [2, 3]. A recent meta-analysis of seventeen retrospective and prospective studies concludes that DBT has higher sensitivity to DM and at least as high specificity [1]. The same result was confirmed in a recent randomized study, in which DBT detected 48 percent more cancers than DM [4]. At the same time, the implementation of DBT in screening has been uneven, depending on country-specific circumstances. For example, the current recommendation from the European Commission’s Initiative on Breast Cancer is conditional, recommending either the use of DBT or DM (but not both) [5]. In contrast, US clinics have been permitted to use DBT in screening since 2011 [6].

To illustrate the regulatory difficulties facing DBT, consider the early US implementation versus the more conservative Swedish approach. As of June 2024, Sweden has not implemented DBT in screening, although it has long been used for follow-up examinations. This more cautious approach is due to a lack of recommendation from the National Board of Health and Welfare, which is the national agency responsible for regulating screening programs in Sweden. In a 2019 assessment, the agency concluded that DBT was a promising technology but that current evidence was insufficient to merit a recommendation due to a lack of data on interval cancer (cancers diagnosed between screening rounds that might have been missed on screening) [7]. The national recommendations were then revised in 2023, but DBT again failed to make the cut, primarily due to a lack of evidence about how DBT affects interval cancer and mortality and, secondarily, the increased resources required to screen with DBT [8].

We believe that Sweden’s decision to not use DBT in screening is ethically problematic. The problem is that new mammography equipment in Sweden (and in other places) is now delivered “DBT-ready,” which means it can be used for both DM and DBT with only minor modifications. For this reason, there are Swedish mammography units that have been using DBT-ready equipment to screen with DM for years, despite the lower sensitivity of DM. In these cases, women are not screened with the most sensitive technology available, leaving potentially dangerous cancers undetected.

To justify the use of a less sensitive form of mammography, we must cite a compensating feature that makes up the difference, such as higher specificity, less overdiagnosis, or the detection of more dangerous tumors. However, we presently lack compelling evidence that DM has such advantages over DBT. Setting aside issues of cost-effectiveness (which will be dealt with later), the most plausible objection to DBT concerns overdiagnosis, as a recent meta-study found no difference in the effect of DM and DBT on interval cancer [9]. However, the evidence regarding interval cancer remains weak: the included studies had not been designed to investigate interval cancers, and no studies have been conducted on repeat screening rounds. Available evidence also suggests that the additional tumors found with DBT are of similar type and grade as those found with DM [10] and might in fact even be more aggressive [11]. It would therefore be surprising if DBT did not have a (perhaps delayed) positive effect on breast cancer mortality.

Resolving these questions may take years, which prompts the question: Why are we not using DBT in the meantime? When the use of a new imaging technology requires clinics to buy new expensive equipment, it might be prudent to wait. But when the equipment is already available, it makes less sense to choose the less sensitive option. A reluctance to use DBT in these situations might, we suggest, rather be explained by what is known as status quo bias, i.e., as a habitual preference for an already implemented technology [12]. Indeed, had DBT been our standard screening method today, it is unlikely to have been replaced by DM, even if the same arguments about, for example, potential overdiagnosis would have applied. More likely, we would, in such circumstances, have continued to use the most sensitive method (i.e., DBT) going forward, while waiting for better data on interval cancer and mortality.

## Comparing DBT with DM+

We suggest the case of DM+ is useful for reducing potential status quo bias when deliberating about the use of DBT in screening. The main difference between the cases is that DM+ is conceptualized as an improvement of DM while DBT is often considered, at least from a regulatory perspective, as a “new” technology. Because we are less likely to conceive of DM+ as a new technology, our reactions to it should be less influenced by status quo bias.

To make our argument more transparent, we will structure it more explicitly. We claim that

1. it would be ethically required to use DM+ equipment without reduced sensitivity in screening and that
2. if it would be ethically required to use DM+ equipment without reduced sensitivity in screening, then it is also ethically required to use DBT-ready equipment for DBT in screening, from which it follows that
3. it is ethically required to use DBT-ready equipment for DBT in screening.

To make this argument as uncontroversial as possible, we will restrict it to circumstances where it is practically feasible to use the equipment. Sufficient reader time has to be available to examine the more time-consuming pseudo-3D images of DBT, and the clinic must have or be able to acquire the required digital infrastructure and storage space. Moreover, the conclusion (3) only concerns the choice between DM and DBT using DBT-ready equipment, and therefore sets aside the question of whether there are other technologies (e.g., MRI, CESM, breast-CT) that are superior to both DBT and DM. Finally, we assume that premise (1) is plausible and that the senior physician’s decision to reduce the image quality of DM+ is unjustified. The argument, then, hangs on whether premise (2) can be successfully defended.

Premise (2) relies on the similarity between implementing DBT and DM+ on available equipment. In both cases we have the option to attain higher sensitivity compared to baseline DM. In neither case do we have strongly supported reasons to believe increased sensitivity comes at the cost of substantially increased overdiagnosis, substantially reduced specificity, or fails to further reduce mortality. To reject premise (2), we must therefore find some other relevant difference between the cases. In what remains of this paper, we consider two potential problems with the analogy. The first is that DBT is a new technology, while the second concerns the cost-effectiveness of DBT.

## New technologies vs improved technologies

As noted previously, DBT is sometimes considered a new technology, while DM+ is more clearly an improvement of an existing technology (i.e., of DM). Could this difference by itself be relevant? We can think of four major ways in which the fact that a technology counts as new might justify us to be more cautious about an implementation, but none of these apply to DBT:

- New technologies may introduce new risks to patients. This is especially true when we switch to a new modality, such as from mammography to MRI. Accordingly, we have more reason to be cautious when the modality itself is unproven. However, DBT does not involve a change in modality per se as it still uses x-rays in comparable doses, and it does not introduce any new risks to patients that were not already present with DM.
- New technologies may require new equipment and procedures, but the equipment might not work as predicted, and the procedures could be flawed. Again, these issues do not arise for DBT, since it uses the same equipment and a similar procedure to DM.
- New technologies may require large capital expenditures to fund new equipment and facilities. However, this is not an issue when implementing DBT on existing DBT-ready equipment. If DBT turns out to be inferior to DM despite current evidence, we can return to using DM with the same equipment.
- New technologies may raise novel ethical issues. For example, information to patients might have to be revised to ensure valid consent, and the distribution of benefits might be altered, leaving some patients worse off. However, patient information can be left mostly intact in a switch to DBT. Additionally, no group is made worse off by the switch.

Even though DBT is considered a new technology, we therefore suggest that in practice and in the context of implementation, it can be treated as an improvement to DM.

## Cost-effectiveness

DBT images take longer to read than DM images and use more storage space, which means that DBT costs more to use than DM. Whether DBT is cost-effective remains an open question: for example, a recent Dutch probabilistic sensitivity analysis says that switching from DM to DBT has a 35% probability of being cost-effective at a willingness-to-pay threshold of €20,000 (the Dutch threshold for cost-effectiveness) per quality-adjusted life-year gained and a 66% chance if the threshold is raised to €35,000 [13].

In our description of DM+, we made no mention of any cost increases. For the sake of the argument, however, we can assume that DM+ images benefit from a higher resolution, which requires readers to spend more time examining magnified details of DM+ images, and which also increases the costs of data storage. This preserves the analogy, but also confronts us with a new question: if DM+ were to increase screening costs just like DBT, would this now justify the senior physician’s decision to reduce its sensitivity?

As far as we can tell, it does not. Women participating in a screening program cannot be expected to forego screening benefits merely because we are uncertain about the cost-effectiveness of using currently existing equipment to detect more potentially dangerous cancers. At the very least, we should offer compelling evidence that DM+ fails to be cost-effective before we reduce its sensitivity. Thus, until health economic studies can point to a clear advantage of downgrading the image quality in such a way, using the unaltered DM+ images appears to be the most sensible approach going forward.

That being said, the economics of DBT remain important to consider in any implementation, especially in the context of a nationally regulated and funded screening program. We should therefore mention two caveats to our discussion, which become important when moving from our more abstract discussion to real-world circumstances.

First, clinics sometimes lack the resources to implement even provenly cost-effective technologies. Similarly, individual clinics may lack the resources required to implement DBT. Although we have suggested there are good ethical reasons to implement DBT using available equipment, whether we choose to act on those reasons is ultimately a political issue.

Second, uncertainty about cost-effectiveness can be a major problem when an implementation requires large up-front investments in new equipment, training of staff, and surrounding infrastructure. That is because such investments may be in vain if the technology turns out to not be cost-effective in the future, becoming what economists would call “stranded assets”. It is therefore important to our argument that the clinics already have much of the necessary equipment to use DBT, including the required digital infrastructure and storage space.

## Conclusion

We have argued that clinics with access to DBT-ready equipment are ethically required to use DBT in screening when practically possible.

## Abbreviations

DBT: Digital breast tomosynthesis
DM: Digital mammography
